# Supplementary material for: Hydroxytyrosol Recovers SARS-CoV-2-PLpro-Dependent Impairment of Interferon Related Genes in Polarized Human Airway, Intestinal and Liver Epithelial Cells
Source: Antioxidants (Basel). 2022 Jul 27;11(8):1466. doi: 10.3390/antiox11081466 (PMC9404978; doi:10.3390/antiox11081466)
Supplement: Supplementary file 1 [file antioxidants-11-01466-s001.zip › antioxidants-1802265-supplementary.pdf]

Supplementary Materials and Data

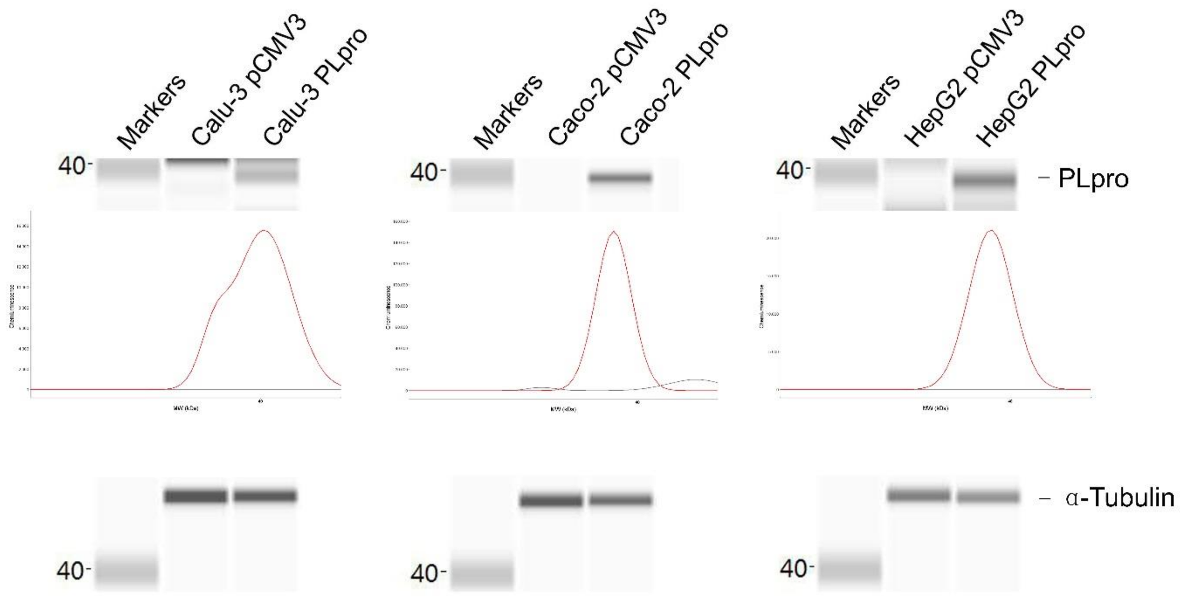

Figure S1. Expression of PLpro protein in epithelial cells. Detection of PLpro in Calu-3, Caco-2, and HepG2 cells by capillary western blot assay. Capillary western blot results for PLpro are shown as gel-like image view in the upper panels, electropherograms in the middle panels, and α-Tubulin as a loading control in the lower panels.

A

Probe code
18S-Hs99999901_s1
GAPDH-Hs99999905_m1
HPRT1-Hs99999909_m1
GUSB-Hs99999908_m1
ARHGEF1-Hs00180327_m1
ARHGEF10-Hs00206020_m1
ARHGEF11-Hs00207600_m1
ARHGEF12-Hs00209661_m1
ARHGEF15-Hs00209087_m1
ARHGEF2-Hs00190884_m1
ARHGEF3-Hs00219609_m1
ARHGEF4-Hs00209700_m1
ARHGEF5-Hs00180533_m1
ARHGEF6-Hs00374462_m1
ARHGEF7-Hs00388776_m1
ARHGEF9-Hs00209514_m1
ATF4-Hs00909568_g1
CREB1-Hs00231713_m1
CREB3-Hs00197255_m1
CREB3L4-Hs00370116_m1
EIF4E-Hs00913390_m1
EIF4EBP1-Hs00607050_m1
FRAP1-Hs00234522_m1
H3F3B-Hs00287906_s1
HIST1H3A-Hs00543854_s1
HIST1H3B-Hs00605810_s1
HIST1H3C-Hs00747483_s1
HIST1H3D-Hs00371415_s1
HIST1H3F-Hs00819633_s1
HIST1H3G-Hs00853299_s1
HIST1H3H-Hs00818527_s1
HIST1H3I-Hs00605800_s1
HIST1H3J-Hs00361917_s1
HIST3H3-Hs00605762_s1
IFNA1-Hs00855471_g1
IFNA14-Hs00533748_s1
IFNA16-Hs03005057_sH
IFNA17-Hs00819693_sH
IFNA2-Hs02621172_s1
IFNA4-Hs01681284_sH
IFNA5-Hs00818220_s1
IFNA6-Hs00819627_s1
IFNA7-Hs01652729_s1
IFNA8-Hs00932530_s1
IFNAR1-Hs01066116_m1
IFNAR2-Hs00174198_m1
IFNB1-Hs00277188_s1
IFNG-Hs00989291_m1
IFNGR1-Hs00166223_m1
IFNGR2-Hs00194264_m1
IFNK-Hs00737883_m1
IFNW1-Hs00357857_s1
IL6-Hs00985639_m1
IRF9-Hs00196051_m1
IRS1-Hs00178563_m1
IRS2;LOC728767-Hs00275843_s1
JAK1-Hs01026983_m1
JAK2-Hs01078136_m1
MAP2K3-Hs00177127_m1
MAP2K6-Hs00992389_m1
MAP3K1-Hs00394890_m1
MAP3K2-Hs00179099_m1
MAP3K3-Hs00176747_m1
MAP3K4-Hs00245958_m1
MAP3K5-Hs00178726_m1
MAP3K6-Hs00177968_m1
MAPK11-Hs00177101_m1
MAPK12-Hs00268060_m1
MAPK13-Hs00234085_m1
MAPK14-Hs00176247_m1
MCF2L-Hs00389840_m1
NET1-Hs00610507_m1
PIK3C2A-Hs00153223_m1
PIK3C2B-Hs00153248_m1
PIK3C2G-Hs00362135_m1
PIK3C3-Hs00176908_m1
PIK3CA-Hs00180679_m1
PIK3CB-Hs00927728_m1
PIK3CD-Hs00192399_m1
PIK3R1-Hs00381459_m1
PIK3R2-Hs00178181_m1
PIK3R3-Hs00177524_m1
PIK3R4-Hs00300795_m1
PIK3R5-Hs00204803_m1
PRKCD-Hs01090047_m1
RAC1-Hs01025984_m1
RPS6-Hs01058685_g1
RPS6KA4-Hs00177670_m1
RPS6KA5-Hs00178054_m1
RPS6KB1-Hs00177357_m1
STAT1-Hs01013989_m1
STAT2-Hs01013123_m1
TYK2-Hs00177464_m1
VAV1-Hs00232108_m1
VAV2-Hs00610104_m1
VAV3-Hs00196125_m1

B

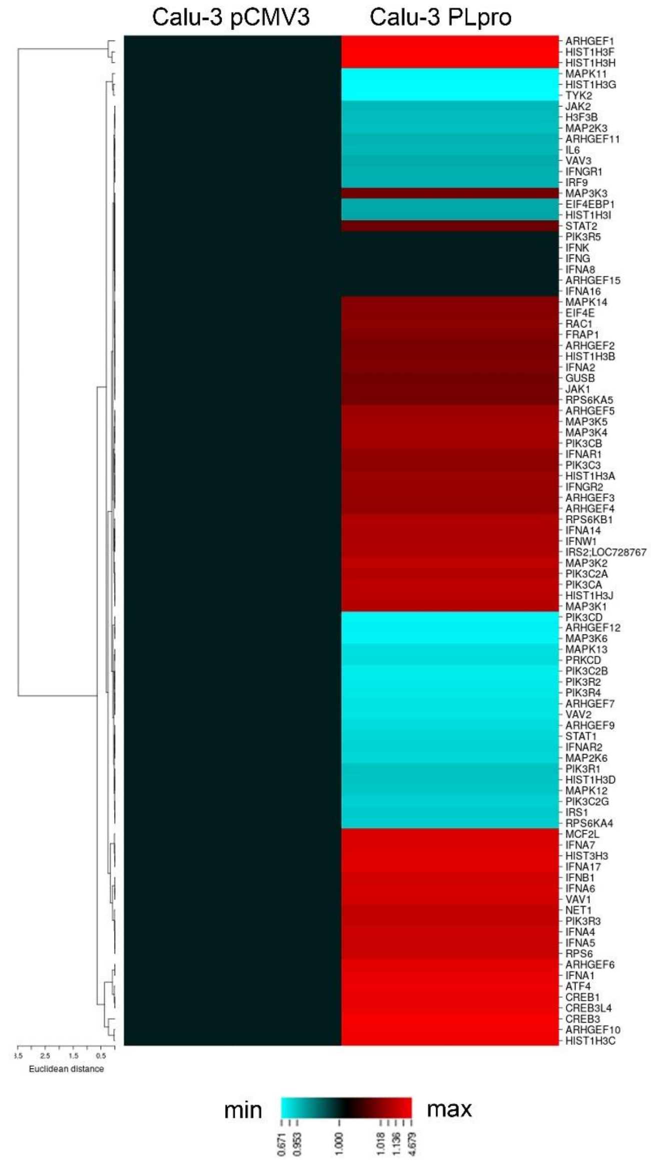

Figure S2. PLpro expression up-regulates genes of IFN pathway. (**A**) List of TaqMan Gene Expression Assays ID for each well on the 96 plate; (**B**) Heatmap representation of interferon-related genes analyzed by TaqMan® Array Human Interferon Pathway that were up-regulated (in red) or down-regulated (in light blue) in Calu-3 cells transfected with plasmid for PLpro (PLpro) or empty vector (pCMV3). Data are the mean ± SD of three independent experiments.

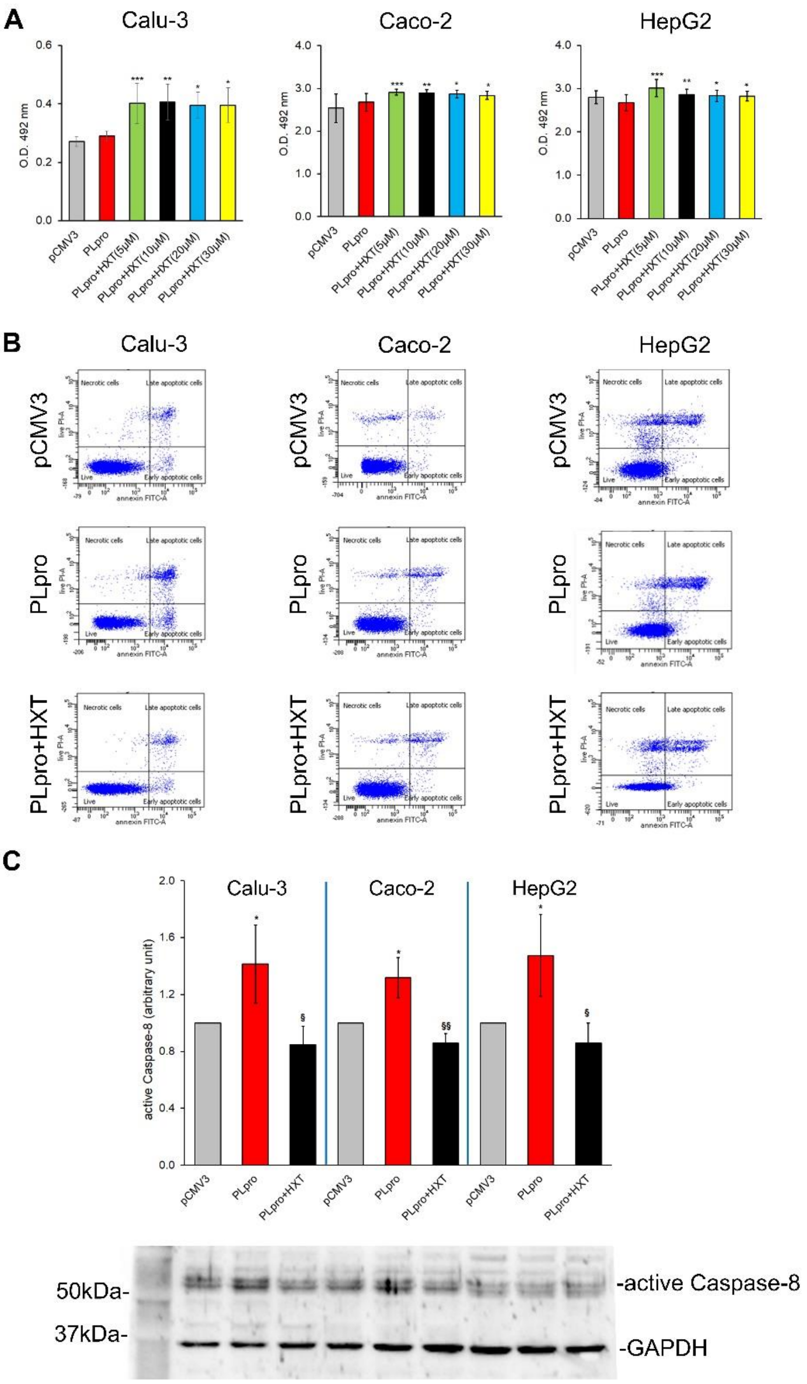

Figure S3. Analysis of apoptosis. (**A**) Cell viability assessed by XTT assay in Calu-3, Caco-2 and HepG2 cells transfected with plasmid for PLpro (PLpro) or empty vector (pCMV3) treated with or without 10 μM HXT for 24 hours. Data refers to mean O.D. of two independent experiments repeated at least in quintupled. Data were analyzed by 2-tailed t tests, **p* <0.05, **p <0.01, ***p <0.001 vs pCMV3. (**B**) Plots show the apoptotic rate of PLpro Calu-3, Caco-2 and HepG2 cells treated with or without 10 μM HXT for 24 hours and compared to untreated (pCMV3) cells measured by FITC Annexin V and flow cytometry. **(C)** Densitometric analysis (*upper panel*) and representative western blotting (*lower panel*) for active Caspase-8 in Calu-3, Caco-2 and HepG2 cells transfected with empty vector (pCMV3), or with plasmid for PLpro (PLpro) subjected or not with 10 μM HXT for 24 hours. GAPDH is reported as loading control. The original image is reported in the supplementary file name blots.

Data were analyzed by 2-tailed *t* tests, *p <0.05 vs pCMV3; § p <0.05, §§ p <0.01 vs PLpro.

A

	A	B	C	D	E	F	G	H	I	J	K	L
1	POS	POS	NEG	NEG	EOTAXIN	EOTAXIN-2	GCSF	GM-CSF	ICAM-1	IFN-γ	I-309	IL-1α
2	POS	POS	NEG	NEG	EOTAXIN	EOTAXIN-2	GCSF	GM-CSF	ICAM-1	IFN-γ	I-309	IL-1α
3	IL-1β	IL-2	IL-3	IL-4	IL-6	IL-6sR	IL-7	IL-8	IL-10	IL-11	IL-12 p40	IL-12 p70
4	IL-1β	IL-2	IL-3	IL-4	IL-6	IL-6sR	IL-7	IL-8	IL-10	IL-11	IL-12 p40	IL-12 p70
5	IL-13	IL-15	IL-16	IL-17	IIP-10	MCP-1	MCP-2	M-CSF	MIG	MIP-1α	MIP-1β	MIP-1δ
6	IL-13	IL-15	IL-16	IL-17	IIP-10	MCP-1	MCP-2	M-CSF	MIG	MIP-1α	MIP-1β	MIP-1δ
7	RANTES	TGF-β1	TNF-α	TNF-β	s TNF RI	s TNF RII	PDGF-BB	TIMP-2	BLANK	BLANK	NEG	POS
8	RANTES	TGF-β1	TNF-α	TNF-β	s TNF RI	s TNF RII	PDGF-BB	TIMP-2	BLANK	BLANK	NEG	POS

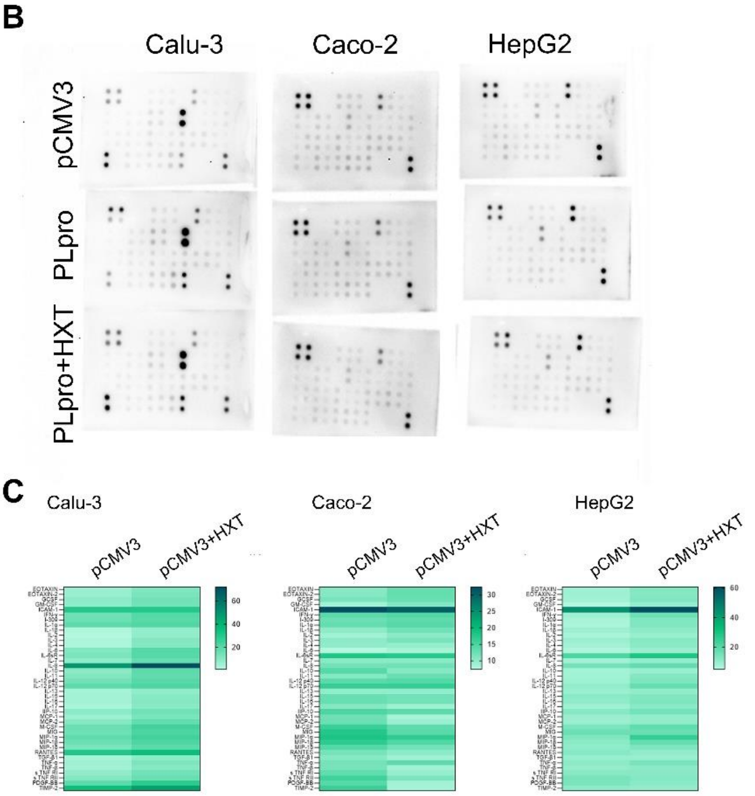

Figure S4. Inflammatory pattern by antibody array. (A) Layout of antibodies as spotted on the membrane (upper panel). The abbreviated names for 40 different cytokine probes are reported. **(B)** Representative photographs of cytokine arrays in which each cytokine is represented by duplicate spots on a single membrane (lower panel). **(C)** Heatmap representation of semi-quantitative expression of 40 pro-inflammatory cytokines and chemokines by antibody array in Calu-3, Caco-2, and HepG2 cells expressing pCMV3 treated or not with HXT.

Table S1. Primers sequences for SYBR Green qRT-PCR.

Genes	Accession number	Sequence 5′ - 3′
**SarsCoV2 PLpro/NSP3**	NC_045512.2(4955-5905nt)	Forward: CAAGGGCTGGTGAAGCTGCTA<br>Reverse: CTTAAGGGTTGTCTGCTGTTGTCC
**IFNA1**	NM_024013.3	Forward: GGAGTTTGATGGCAACCAGT<br>Reverse: CTCTCCTCCTGCATCACACA
**IFNA7**	NM_021057.2	Forward: CAGACCCACAGCCTGCGT<br>Reverse: AAACTCCTCCTCTGGGAATCTG
**ATF4**	NM_182810.3	Forward: TCAAACCTCATGGGTTCTCC<br>Reverse: GTGTCATCCAACGTGGTCAG
**CREB3**	NM_006368.5	Forward: TTGCTAGGCTAGTACTGACAGATGA<br>Reverse: ATTTCAAGACCCTGCTCTCTAAACC
**CREB3L4**	NM_001255978.2	Forward: CTCAAGAAGGTCAGGAGGAAAATCC<br>Reverse: CTGAGCTACCAAGGAGATGTTGTG
**HIST3H3**	NM_003493.3	Forward: GAGCTGCTAATCCGCAAGTT<br>Reverse: TGGATGACACACAGGTTGGT
**HIST1H3F**	NM_021018.3	Forward: AGCTACTGATTCGCAAGCTACC<br>Reverse: ACAGGTTGGTGTCCTCAAAGAG
**HIST1H3H**	NM_003536.3	Forward: GATCAGAAAGCTGCCTTTTCAG<br>Reverse: GATGTCCTTGGGCATGATAGTC
**MCF2L**	NM_015205.3	Forward: ATGAAATCATGCACCAGGACAT<br>Reverse: CATGACATTCTGGAACTCCTTG
**TNF-α**	NM_000594.4	Forward: CCGAGTGACAAGCCTGTAGC<br>Reverse: AGGAGGTTGACCTTGGTCTG
**IL-6**	NM_000600.5	Forward: AGACAGCCACTCACCTCTTCAG<br>Reverse: TTCTGCCAGTGCCTCTTTGCTG
**IL1-β**	NM_000576.3	Forward: GCTGAGGAAGATGCTGGTTC<br>Reverse: TCCATATCCTGTCCCTGGAG
**GAPDH**	NM_002046.7	Forward: TCCAAAATCAAGTGGGGCGA<br>Reverse: TGATGACCCTTTTGGCTCCC

Table S2. Means of raw data for pro-inflammatory protein array.

	Calu-3		Caco-2		HepG2	
	Mean pCMV3	*Mean PLpro*	*Mean pCMV3*	*Mean PLpro*	*Mean pCMV3*	*Mean PLpro*
**EOTAXIN**	6.232	11.203	10.520	12.958	8.328	12.806
**EOTAXIN-2**	6.358	11.680	10.674	13.040	8.251	12.455
**GCSF**	8.695	11.774	11.557	12.774	7.906	13.015
**GM-CSF**	8.172	9.235	8.592	10.860	5.087	9.869
**ICAM-1**	30.616	30.528	32.398	30.451	44.419	60.744
**IFN-γ**	14.789	12.654	13.708	14.407	8.337	16.236
**I-309**	15.390	12.532	12.577	13.506	6.886	13.066
**IL-1α**	18.798	15.320	13.039	13.963	6.218	13.119
**IL-1β**	1.978	7.938	10.089	12.23	8.619	11.243
**IL-2**	1.210	5.603	8.571	10.264	6.630	9.157
**IL-3**	2.441	7.7	10.235	11.622	8.994	12.125
**IL-4**	2.335	7.097	9.839	9.564	7.684	11.313
**IL-6**	10.919	16.342	7.458	8.4525	5.787	9.021
**IL-6sR**	12.284	13.358	17.948	17.364	21.323	28.872
**IL-7**	8.194	8.4845	10.295	10.411	7.710	10.213
**IL-8**	48.953	62.727	11.916	12.169	15.038	17.671
**IL-10**	9.219	7.134	14.072	10.619	7.349	9.097
**IL-11**	11.290	9.686	10.578	12.756	7.832	12.647
**IL-12 p40**	14.259	12.007	13.758	14.306	8.320	13.711
**IL-12 p70**	18.657	16.880	16.392	16.477	10.266	17.258
**IL-13**	3.856	5.502	9.795	9.739	9.233	10.246
**IL-15**	4.407	8.15	12.739	12.084	11.495	12.967
**IL-16**	4.536	6.231	12.824	11.925	10.792	13.378
**IL-17**	2.992	5.276	10.615	8.815	8.181	11.502
**IIP-10**	9.344	8.464	14.604	13.633	12.038	15.110
**MCP-1**	5.839	8.721	12.289	8.309	8.957	9.938
**MCP-2**	10.208	8.763	12.587	8.974	9.051	10.534
**M-CSF**	15.744	13.903	16.016	13.320	13.537	18.214
**MIG**	14.966	13.864	17.596	13.847	16.297	16.691
**MIP-1α**	18.111	16.136	18.356	16.909	13.076	25.018
**MIP-1β**	15.373	13.014	16.865	14.398	11.443	17.175
**MIP-1δ**	12.546	11.107	14.182	12.015	10.643	13.496
**RANTES**	30.834	61.17	11.591	10.209	12.084	10.323
**TGF-β1**	3.378	5.151	10.541	8.5385	9.406	9.423
**TNF-α**	5.166	7.374	11.857	13.610	11.286	14.594
**TNF-β**	4.620	6.196	11.708	8.5425	9.961	11.357
**s TNF RI**	8.007	8.527	13.194	9.473	11.862	11.812
**s TNF RII**	10.866	9.305	15.526	11.441	14.356	12.311
**PDGF-BB**	18.442	13.364	14.0605	8.544	13.820	11.504
**TIMP-2**	35.236	43.716	16.01125	8.485	10.991	10.332
